# Volatile Oils of *Nepeta tenuifolia* (Jing Jie) as an Alternative Medicine against Multidrug-Resistant Pathogenic Microbes

**DOI:** 10.1155/2018/8347403

**Published:** 2018-04-11

**Authors:** Yi-Hsuan Lee, Chao-Min Wang, Po-Yu Liu, Ching-Chang Cheng, Zong-Yen Wu, Shu-Ying Tseng, Kwong-Chung Tung

**Affiliations:** ^1^Department of Veterinary Medicine, College of Veterinary Medicine, National Chung-Hsing University, Taichung, Taiwan; ^2^Research Center for Biodiversity, China Medical University, Taichung, Taiwan; ^3^Department of Internal Medicine, Taichung Veterans General Hospital, Taichung, Taiwan; ^4^Rong Hsing Research Center for Translational Medicine, National Chung Hsing University, Taichung, Taiwan; ^5^Laboratory Animal Service Center, Office of Research and Development, China Medical University, Taichung 40402, Taiwan

## Abstract

Essential oils from the dried spikes of *Nepeta tenuifolia* (Benth) are obtained by steam distillation. Pulegone was identified as the main component in the spikes of *N. tenuifolia* through analysis, with greater than 85% purity obtained in this study. The essential oils are extremely active against all Gram-positive and some Gram-negative reference bacteria, particularly *Salmonella enterica*, *Citrobacter freundii*, and *Escherichia coli*. The minimum inhibitory concentration was found to be between 0.08 and 0.78% (against *S. enterica*), 0.39 and 0.78% (against *C. freundii*), and 0.097 and 0.39% (against *E. coli*), whereas the minimum bactericidal concentration varied in range from 0.097% to 1.04%. In general, the essential oils show a strong inhibitory action against all tested reference strains and clinical isolates. However, the antibacterial activity of EOs against both *Pseudomonas aeruginosa* reference strains and clinical isolates was relatively lower than other Gram-negative pathogens. The essential oils of *N. tenuifolia* also displayed bactericidal activities (MBC/MIC < 4) in this study. These findings reflect the bactericidal activity of the essential oils against a wide range of multidrug-resistant clinical pathogens in an in vitro study. In addition, we propose the fragmentation pathways of pulegone and its derivatives by LC-ESI-MS/MS in this study.

## 1. Introduction

Although plants lack an immune system, they produce defensive chemicals to attack invading organisms. They can resist bacterial attacks through several defense mechanisms. Extensive research uncovered that the defensive substances belong to a wide range of different chemical classes such as flavonoids, terpenoids, alkaloids, phenolic acids, essential oils, and polyphenols [1]. As a result of these studies, there has been an increased interest in the use of natural substances as alternative medicines against multidrug-resistant (MDR) microorganisms. MDR microorganisms are widely found in hospitals and are increasingly being isolated from different infections [2, 3].

Lamiaceae, also known as the mint family, has been used over the years in traditional medicine. Many members of this family are of great medicinal value as natural sources of active compounds such as phenolic compounds and essential oils [4]. Essential oils (EOs) are a complex natural mixture of volatile secondary metabolites, which can be isolated from plants by steam distillation. A number of pharmacological studies have been carried out within the mint family and confirm both the antiviral and antimicrobial activities [5–7]. *Schizonepeta tenuifolia* Briq. is a synonym for *Nepeta tenuifolia* (Benth) (also known as Jing Jie in Chinese medicine), which belongs to the Lamiaceae family. A few studies have explored the dry aerial parts of *N. tenuifolia* (Benth), which contain 0.5–1.8% of volatile oils [8]. The traditional use of plants has provided important information that *N. tenuifolia* (Benth) could be used for specific purposes in inflammatory responses, along with the treatment of infectious diseases in traditional Chinese medicine. For example, it is used for treating the symptoms of common influenza such as headache, fever, infections, and sore throat.

Thus, the aim of this study was to evaluate the EOs of *N. tenuifolia* as natural antimicrobials against MDR microorganisms, which are some of the principal clinical pathogens causing skin diseases in pets. Moreover, we determined the profiles of MIC, MBC, and the MBC/MIC ratio on 16 different reference strains of the microorganisms.

## 2. Materials and Methods

### 2.1. Plant Material

**Figure 1:**
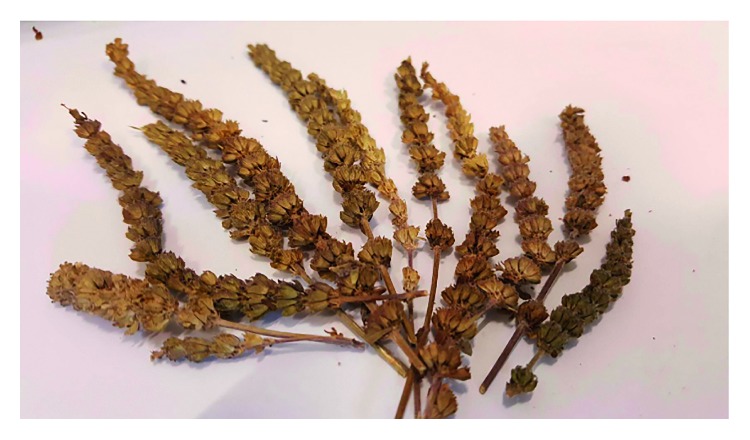
Dried terminal flower spike samples of *Nepeta tenuifolia*.

Terminal flower spikes from the medicinal plant *N. tenuifolia* were used in this study. Dried samples of *N. tenuifolia* were purchased from a Chinese herbal medicine pharmacy in Taichung, Taiwan (Figure 1). The samples were identified by Dr. Hsien-Cheh Chang. A voucher specimen has been deposited in the Lifu collection room of Chinese Medicine, Taichung (Chao 0111).

### 2.2. Isolation of the Essential Oils

All dried spike part samples of *N. tenuifolia* were cut into small pieces. The samples (600 g) were then extracted using pure water as a solvent (1000 ml) at 60–70°C under vacuum concentration. The obtained oils were stored as stock in sealed glass vials at 4°C in a refrigerator.

### 2.3. Analysis of the Essential Oils

**Figure 2:**
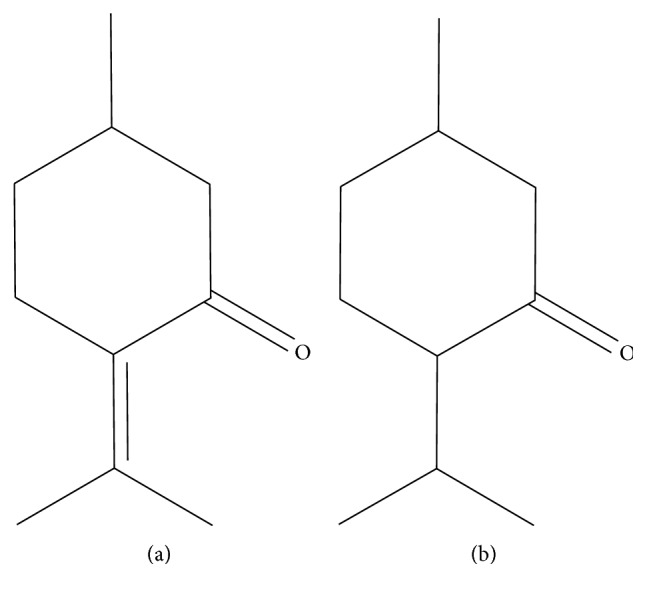
Chemical structure of pulegone (a) and menthone (b).

The analysis of the compounds was done according to the method reported previously [8–10], with a slight modification. Briefly, analytical liquid chromatography-electrospray ionization/tandem mass spectrometry (LC-ESI-MS/MS) was carried out in a high-pressure liquid chromatograph (UltiMate 3000, Dionex) equipped with a mass spectrometer (HCT Ultra PTM Discovery System, Bruker Daltonics) and an Atlantis T3 RP-18 column (150 mm × 2.1 mm, 3 *μ*m; Waters, USA). The column was eluted with buffer A (5% acetonitrile with 0.1% formic acid) and buffer B (100% acetonitrile with 0.1% formic acid) at a flow rate of 0.25 ml/min at 25°C. The solvent extraction was performed as follows: 0 to 60% buffer B over the first 5 min, 60 to 80% buffer B over the next 15 min, 80 to 95% buffer B over the next 10 min, isocratic for 5 min, and 0% buffer B for the final 10 min. The injection volume was measured at 5 *μ*l in each case. Both positive and negative ionization mode MS analyses were performed. The temperature of the ion source was maintained at 100°C, while the drying temperature was 365°C, and the desolvation gas N_2_ had a flow rate of 12 L/min. Production scans for mass were performed using low-energy collision (20 eV), with argon being used as the collision gas. The analysis was initially carried out through the use of full-scan, data-dependent MS/MS, from *m*/*z* 50 to *m*/*z* 1,000. The molecular ion peaks and mass spectra were compared with those of reference compounds. (+)-Menthone (≥98.5%) and (*S*)-(−)-pulegone (98%) (Figure 2) were purchased from Sigma-Aldrich and were used as marker compounds. The obtained data were further processed by using Bruker Daltonics Data Analysis Software (version 4.0). All tests and analyses were run in triplicate and then averaged.

### 2.4. Microbial Strains

The studied bacteria included both reference strains (from the American Type Culture Collection and Bioresource Collection and Research Center, Taiwan) and clinical strains. The antimicrobial activity of the EOs of the standard samples was tested against 16 different reference strains of the microorganisms in total. The eleven Gram-negative bacterial strains included *Citrobacter freundii* (BCRC 12291 and BCRC 12292), *Escherichia coli* (BCRC 10675 and ATCC 43890), *Salmonella enterica* subsp. (BCRC 10744 and BCRC 10747), and *Pseudomonas aeruginosa* (ATCC 15692, BCRC 10944, ATCC 9027, ATCC 27853, and BCRC 13053). In addition, five tested Gram-positive bacterial strains, including *Bacillus cereus* (BCRC 10603), *Listeria monocytogenes* (BCRC 14846), and *Staphylococcus aureus* subsp. (BCRC 12657, ATCC 29213, ATCC 25923), were used in this study.

A total of 20 clinical isolates of common pathogenic bacteria (*E. coli*, *P. aeruginosa*, and *S. pseudintermedius*) known to have drug susceptibility were maintained in the Microbiology Laboratory of the Veterinary Hospital of National Chung Hsing University, Taiwan. These strains that were selected for use included both susceptible and resistant strains for each antibiotic.

### 2.5. Susceptibility Tests and Determination of MIC and MBC

A 5% glucose and a 1% phenol red-supplemented nutrient broth were pipetted into each well as outlined previously [11] after a slight modification in order to assess the minimal inhibitory concentration (MIC) of the essential oil.

Fifty microliters of 0.5 McFarland standard bacterial suspension was then added to each well, with the plates incubated at 37°C for 24 hours. Visual observation of growth was based on the color change of the phenol red indicator from red to yellow, reflecting acid waste being produced due to the growth of the microorganism.

The concentrated extract was mixed with Tween 80 and then added to the broth through serial twofold dilution. The microdilution was performed in 96-well plates. The final concentrations ranged from 12.5%, 6.25%, 3.125%, 1.5625%, 0.7813%, 0.3906%, 0.1953%, 0.0976%, to 0.0485%. Only Tween 80 diluted with broth was used in this study as a negative control. After 24 hours of incubation time at 37°C, the well displaying the least growth was considered to be the first well, while the corresponding concentration was considered to be the minimum bactericidal concentration (MBC). The MBC was detected through subculturing onto a Müller–Hinton agar (Merck, Darmstadt, Germany). The agar plates were incubated at 37°C overnight for 24 hours, and on the next day, readings were taken. All tests were performed in triplicate.

### 2.6. Chemicals for Antimicrobial Assays

The eight antibiotic discs (Oxoid, UK) used included aminoglycosides represented by gentamicin (10 *μ*g) and fluoroquinolones represented by enrofloxacin (5 *μ*g). Additionally, penicillins were represented by both amoxicillin/clavulanic acid (30 *μ*g) and ampicillin (10 *μ*g), whereas cephalosporins were represented by both cefazolin (30 *μ*g) and cephalexin (30 *μ*g). Finally, chloramphenicol (30 *μ*g) and doxycycline (30 *μ*g) as a tetracycline antibiotic were also tested. Based upon the diameter of clear zones around the antibiotic discs and the Clinical and Laboratory Standards Institute (CLSI, 2014) guidelines, the strains were categorized as either sensitive (S) or resistant (R) to the drug. Intermediate susceptibility was regarded as resistant (R) in our experimental setting.

## 3. Results

### 3.1. Analysis of the Essential Oils

**Figure 3:**
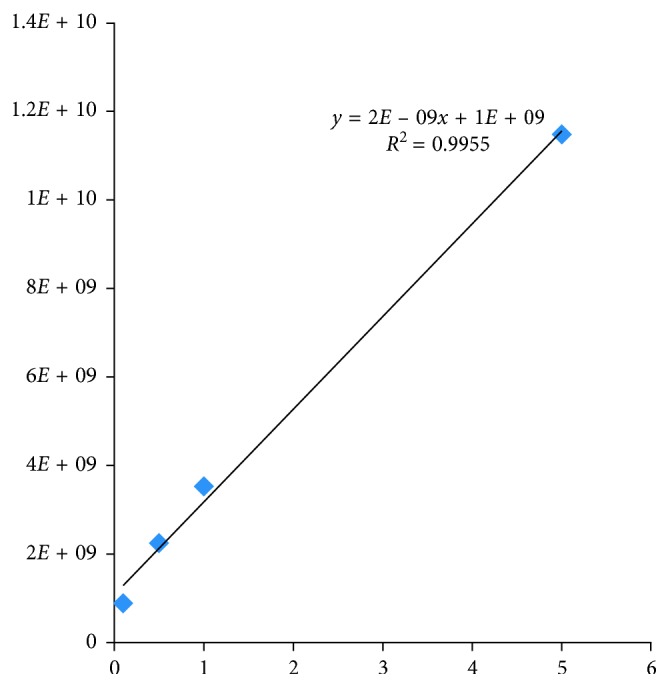
Determination of pulegone concentration by comparing the standard samples.

The steam distillation of the dried spike parts of *N. tenuifolia* provided clear, light yellow liquid-like oils with a yield of 1.67% (v/w), calculated on the basis of dry weight. Here, the quantification of compounds was performed using ion-selective monitoring to determine the specific parent ion of both pulegone (152.9 *m*/*z* [M + H]^+^) and menthone (155.0 *m*/*z* [M + H]^+^). EOs were freshly prepared at 1% concentration, while reference compounds were prepared at a concentration range of 0.01–10%. Results show that all target compounds can provide higher precursor ions [M + H]^+^ in the positive ion mode than in the negative one, when they are under full-scan mode. Therefore, the positive mode was selected for both pulegone and menthone detection. Furthermore, the EOs of *N. tenuifolia* were also analyzed for menthone and pulegone through LC-ESI-MS techniques and then compared with previous studies in order to identify the EOs profile in this study. The amount of pulegone in the EOs of *N. tenuifolia* was detected as 84.9% ± 2.9%. For pulegone, favorable linearity of the calibration curves was achieved with the mean values for the regression equation *y*=2*E*+09*x*+1*E*+09(*R*^2^=0.9955). All analyses were performed in triplicate (Figure 3).

**Figure 4:**
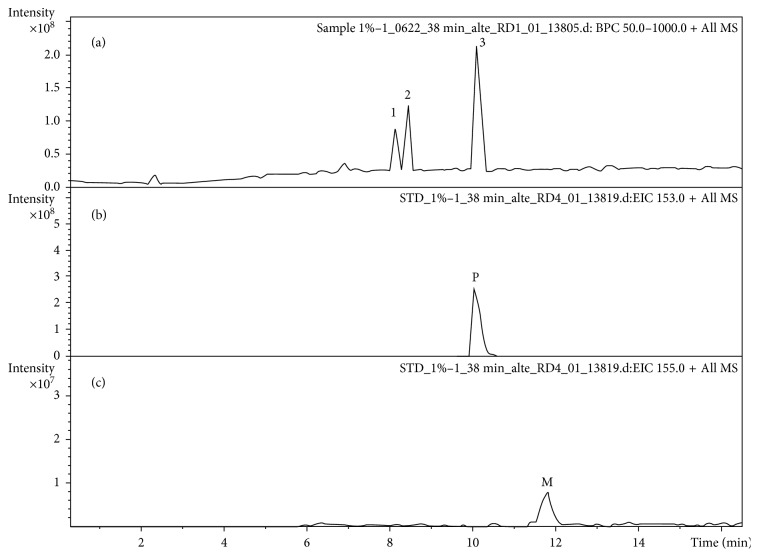
The LC-MS chromatograms of sample (a), pulegone (b), and menthone (c) in positive ion mode.

**Figure 5:**
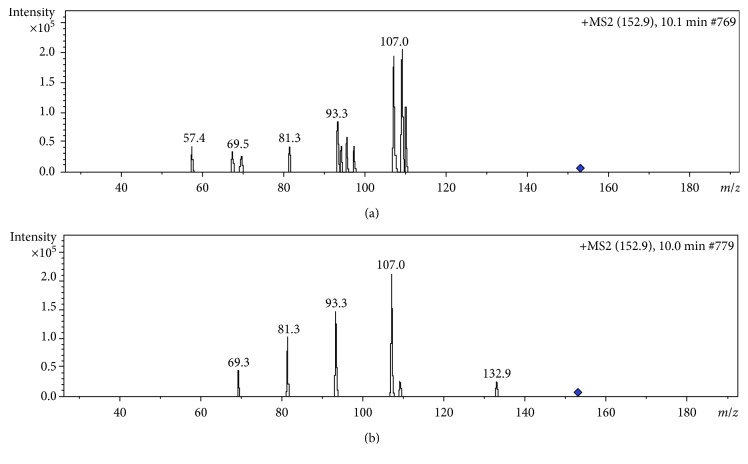
MS/MS spectra of sample (a) and pulegone (b).

**Figure 6:**
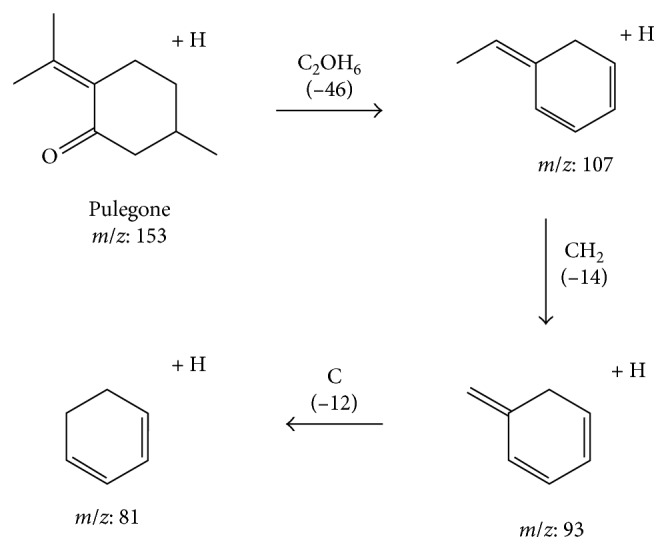
Fragmentation pathways of pulegone and its derivatives.

As shown in Figure 4, there are three major peaks in the extract, with the major component of the EOs being peak 3. By comparing the retention time and mass spectra with those of standard compounds, peak 3 was identified as pulegone; however, menthone was not detected in the extract obtained in this study (Figures 4 and 5). Pulegone can form a protonated molecular ion with *m*/*z* 153 [M + H]^+^ in the positive ion mode. Here, it formed *m*/*z* 107 fragment ions when it lost C_2_OH_6_ (*m*/*z* 46). It then formed *m*/*z* 93 fragment ions when it lost CH_2_ (*m*/*z* 14). The proposed fragmentation patterns are shown in Figure 6.

### 3.2. Antimicrobial Assays: Minimal Inhibitory Concentration (MIC), Minimum Bactericidal Concentration (MBC), and MBC/MIC Ratio

Our results showed that the essential oils of *N. tenuifolia* have effective bactericidal activity. This indicated that Gram-positive bacteria are the most sensitive, particularly *B. cereus*, *L. monocytogenes*, and *S. aureus* subsp. The Gram-negative bacterial species inhibited by the extracted oils included *C. freundii*, *E. coli* (BCRC 10675 and ATCC 43890), *S. enterica* subsp., and some *P. aeruginosa* strains (Table 1).

In general, the EOs presented a strong inhibitory action against all tested reference Gram-positive bacteria. The MIC and MBC values are shown in Table 1. Compared to Gram-positive bacteria, Gram-negative reference pathogens are also sensitive to EOs in this study. Regarding *P. aeruginosa*, the EOs displayed low activity in this study, including both reference and clinical isolates. The strains of *P. aeruginosa*, identified as ATCC 15692 and BCRC 10944, showed a strong resistance to the *N. tenuifolia* extract. The extracted oils were only mildly effective against the ATCC 9027 and BCRC 13053 strains of *P. aeruginosa*. The essential oils of *N. tenuifolia* exhibited a strong inhibitory effect against the strain *P. aeruginosa* ATCC 27853 (Table 1).

The antibiotic resistance patterns for clinical isolates are shown in Tables 2–4. Among the strains, seven clinical isolates of *E. coli* were tested in this study, with three being resistant to 6 antibiotics, that is, multidrug-resistant (MDR) isolates. Two *E. coli* strains were fully sensitive, while the remaining two isolates were at least resistant to one or two antibiotics (Table 2). With regard to identifying the effect of EOs on *E. coli*, the MIC and MBC were at 0.163 to 0.781% and showed that all clinical isolates of *E. coli* were strongly susceptible to the *N. tenuifolia* EOs. In addition, the antibiotic resistance pattern of five *S. pseudintermedius* clinical isolates is shown in Table 4. Three isolates were MDR pathogens and two were resistant to ampicillin. All the evaluated clinical isolates of this species presented a strong sensitivity to EOs during antimicrobial activity (Table 4). Of the twenty clinical isolates in total, *E. coli* and *S. pseudintermedius* were the most sensitive to treatment with the EOs in our study. The findings on the effect the EOs have on *P. aeruginosa* clinical isolates vary according to the strain in this study (Table 3). Four isolates of *P. aeruginosa* were observed in which MBC exceeded the highest concentration of the drug in the test medium. The results of the MIC and MBC tests indicated that EOs exhibit a low potential of antibacterial activity against 8 tested clinical isolates. All of the *P. aeruginosa* clinical isolates were multidrug resistant to antibiotics and also resistant to the oils in the present study.

Further details are outlined in Tables 1–4. The antibacterial effect of the EOs was compared according to MBC/MIC values. A collection of 16 reference strains and 20 clinical isolates was used to determine EOs tolerance in this experiment. Susceptibility rates were calculated based on the MBC/MIC values, as bactericidal activity has been defined as the ratio of MBC to MIC at <4 [12]. The activities of *N. tenuifolia* extract oils on reference strains show bactericidal effects as shown in Table 1. Full sensitivity to EOs was observed for all *E. coli* isolates which had MBC/MIC ratios of 1 (Table 2). Three *P. aeruginosa* clinical isolates had MBC/MIC ratios which were not calculable, showing that MIC or MBC exceeded the highest concentration of the drug (Table 3). The oil shows weak activity on the rest of the isolates, with MIC or MBC approaching nearly 12.5%. Moreover, the *N. tenuifolia* extracted oils exhibited MBC/MIC ratios ranging between 1.5 and 4 on *S. pseudintermedius*, as shown in Table 4.

## 4. Discussion

In this study, we have described the MBC and MIC and calculated the MBC/MIC ratio for the susceptibility testing of *N. tenuifolia* extract oils on a wide range of clinically relevant pathogens. The activity of *N. tenuifolia* oils can also likely be due to pulegone, which is the oil's predominant component (the total amount of pulegone is approximately 85%) in our study (Figure 6). Pulegone (Figure 2) is naturally occurring and is classified as a ketone monoterpene, similarly to the way carvone is. It can be obtained from a variety of plants such as the *Mentha* species [13–15]. In a previous study, pulegone exhibited high antimicrobial activity, particularly against *C. albicans* and *S. typhimurium* [16]. Some studies support that monoterpenes may cross the cell membranes, penetrate the interior of the cell, and interact with intracellular sites critical for antibacterial activity [17–19].

The herb *N. tenuifolia* contains volatile oils which were identified mostly as menthone and *R*-(+)-pulegone in previous studies [20, 21]. In our study, we did not detect menthone (Figures 3 and 4). Menthone is a common volatile compound in Lamiaceae. Menthone in peppermint oil is one among the constituents that are present at relatively higher concentration in *Mentha* species, which can also be active against a large number of bacteria such as *E. coli*, *S. aureus*, and *E. faecalis* [22, 23]. A study reported that the different drying methods affected strongly the EO composition of peppermint oil and caused significant reduction of menthone concentration [24]. In addition, the harvesting seasons and growing regions affect the quality of the compounds [25]. It may explain why we did not detect menthone in our study. However, it needs further study of the subject.

Historically, EOs have been used from as far back as ancient times for their medicinal properties. Various EOs and their components possess different pharmacological effects, such as anti-inflammatory, antioxidant, and antimicrobial properties [16, 22, 24, 26, 27]. The traditional use of plants has provided important information that *N. tenuifolia* should be used for specific purposes in anti-inflammatory responses and the treatment of infectious diseases [28]. Previous antibacterial studies have also shown that Lamiaceae EOs, including those from basil, mint, rosemary, sage, and hyssop, are active against *E. coli*, *S. aureus*, *B. cereus*, and *Salmonella* spp. but remain less effective against *Pseudomonas* spp. [29–31]. Our results also correlate with several studies which have shown that the EOs of *Mentha* spp. or other volatile constituents were more responsive to Gram-positive pathogens in the reduction or control of pathogenicity than Gram-negative pathogens [32–34]. The reference strains of *P. aeruginosa* ATCC 15692 and BCRC 10944 and MDR *P. aeruginosa* clinical isolates in our study display a less observed sensitivity to *N. tenuifolia* extract oils (Tables 1–4).

For bactericidal drugs, the MBC/MIC values are less than or equal to 4, whereas an MBC/MIC ratio ≥4 indicates bacteriostatic activity [12, 35]. In this study, most of the MBC/MIC ratios were less than 4, indicating a bactericidal effect that EOs have against reference strains *B. cereus*, *L. monocytogenes*, *S. aureus* subsp. *aureus*, *C. freundii*, *E. coli*, and *S. enterica* subsp. and clinical isolates *E. coli* and *S. pseudintermedius*. The ratios of MBC to MIC for *N. tenuifolia* extract oil for three *P. aeruginosa* clinical isolates (101-190-1, 101-204-2, and 102-336) were listed as “NA” (not calculable), when MIC or MBC exceeded the highest concentration of drug in the test medium, suggesting that the oils may be bacteriostatic or ineffective on *P. aeruginosa.* Taken together, the results of MIC, MBC, and MBC/MIC ratio demonstrate an excellent bactericidal effect on Gram-positive and some Gram-negative pathogens. Most *P. aeruginosa* exhibit higher rates of resistance, and this may be due to rich hydrophobic lipopolysaccharide (LPS) present in the outer membrane, which can provide protection against different agents, thereby limiting the diffusion of hydrophobic compounds through it, while this extra complex membrane is absent in Gram-positive bacteria [36]. In fact, the activity of the EOs would be expected to relate to the structural features and functional groups of the bacterial membranes and the active constituents of the EOs. This results in alterations in the membrane permeability of bacteria, along with the leakage of intracellular materials [37]. These outcomes might also explain why *N. tenuifolia* EOs showed antimicrobial activity in this study. However, there are certain limitations, and future studies should explore the mechanism of *N. tenuifolia* EOs.

## 5. Conclusion

The present work shows that *N. tenuifolia*'s dried aerial parts (spikes and stems) display favorable antimicrobial effectiveness on multidrug-resistant pathogens, particularly more for Gram-positive pathogens than Gram-negative pathogens, in the reduction or control of pathogenicity. In addition, we propose the fragmentation pathways of pulegone and its derivatives by LC-ESI-MS/MS in this study for determining the structural information of the molecule. The extract oils exhibited strong antibacterial activity, with the activity of *N. tenuifolia* EOs being likely attributed to the oil's predominant component (the total amount of pulegone is approximately 85%), although additional future studies are necessary to confirm this theory.

## Figures and Tables

**Table 1:** Minimum inhibitory concentration (MIC), minimum bactericidal concentration (MBC), and MBC/MIC ratios of *Nepeta tenuifolia* extract (%).

Strain designation	MIC (%)	MBC (%)	MBC/MIC	Amp MIC (*μ*g/ml)	Amp MBC (*μ*g/ml)
Gram-positive bacteria					
*Bacillus cereus* (BCRC 10603)	0.195	0.260	1.333	128	128
*Listeria monocytogenes* (BCRC 14846)	0.390	1.170	3.000	0.06	0.06
*Staphylococcus aureu*s subsp. *aureus* (BCRC 12657)	0.780	2.350	3.013	0.06	0.12
*Staphylococcus aureus* subsp. *aureus* (ATCC 29213)	0.780	1.560	2.000	0.12	0.25
*Staphylococcus aureus* subsp. *aureus* (ATCC 25923)	1.560	4.690	3.006	0.06	0.12
Gram-negative bacteria					
*Citrobacter freundii* (BCRC 12291)	0.780	1.040	1.333	32	32
*Citrobacter freundii* (BCRC 12292)	0.390	0.390	1.000	64	64
*Escherichia coli* (BCRC 10675)	0.390	0.390	1.000	8	8
*Escherichia coli* (ATCC 43890)	0.097	0.097	1.000	8	8
*Salmonella enterica* subsp. *enterica* (BCRC 10744)	0.081	0.097	1.200	2	2
*Salmonella enterica* subsp. *enterica* (BCRC 10747)	0.780	0.780	1.000	4	4
*Pseudomonas aeruginosa* (ATCC 15692)	12.500	>12.5	NA	>128	>128
*Pseudomonas aeruginosa* (BCRC 10944)	10.417	12.500	1.200	>128	>128
*Pseudomonas aeruginosa* (ATCC 9027)	5.210	5.210	1.000	>128	>128
*Pseudomonas aeruginosa* (ATCC 27853)	0.780	0.780	1.000	>128	>128
*Pseudomonas aeruginosa* (BCRC 13053)	6.250	8.333	1.333	>128	>128

NA: undetermined because MIC and/or MBC values were under the lower limit of each test range.

**Table 2:** Antibacterial activity of *Nepeta tenuifolia* extracts against *E. coli* clinical isolates and their antibiotic resistance profile.

Strain designation	MIC (%)	MBC (%)	MBC/MIC	Antibiotic resistance pattern^∗^	Amp MIC (*μ*g/ml)	Amp MBC (*μ*g/ml)
E 103-167	0.781	0.781	1	Do	2	2
E 103-203	0.391	0.391	1	Amo, Amp, Cef, Cep, Enf, and Gent	32	32
E 103-187	0.651	0.651	1	Nonresistance	1	1
E 102-272	0.391	0.391	1	Amo, Amp, Cef, Cep, Cham, Do, and Enf	32	32
E 102-346	0.195	0.195	1	Amo, Amp, Cef, Cep, Enf, and Gent	32	32
E 101-051	0.195	0.195	1	Cep and Do	2	2
E 102-231-3	0.163	0.163	1	Nonresistance	2	2

^∗^Amo, amoxicillin; Amp, ampicillin; Cef, cefazolin; Cep, cephalexin; Cham, chloramphenicol; Do, doxycycline; Enf, enrofloxacin; Gent, gentamicin.

**Table 3:** Antibacterial activity of *Nepeta tenuifolia* extracts against *P. aeruginosa* clinical isolates and their antibiotic resistance profile.

Strain designation	MIC (%)	MBC (%)	MBC/MIC	Antibiotic resistance pattern^∗^	Amp MIC (*μ*g/ml)	Amp MBC (*μ*g/ml)
101-190-1	12.500	>12.5	NA	Amo, Amp, Cef, Cep, Cham, and Do	128	128
102-249	12.500	12.5	1	Amo, Amp, Cef, Cep, Cham, Do, and Enf	128	128
101-204-2	>12.5	>12.5	NA	Amo, Amp, Cef, Cep, Cham, and Do	>128	>128
101-230	12.500	12.5	1	Amo, Amp, Cef, Cep, Cham, and Do	128	128
101-189	8.333	12.5	1.5	Amp, Cham, Do, and Gent	128	128
101-319	6.250	12.5	2	Amo, Amp, Cef, Cep, and Gent	>128	>128
102-336	12.500	>12.5	NA	Amo, Amp, Cef, Cep, Cham, Do, Enf	>128	>128
102-370-1	10.417	12.5	1.2	Amo, Amp, Cef, Cep, Cham, Do, and Enf	128	128

^∗^Amo, amoxicillin; Amp, ampicillin; Cef, cefazolin; Cep, cephalexin; Cham, chloramphenicol; Do, doxycycline; Enf, enrofloxacin; Gent, gentamicin; NA: undetermined because MIC and/or MBC values were under the lower limit of each test range.

**Table 4:** Antibacterial activity of *Nepeta tenuifolia* extracts against *Staphylococcus pseudintermedius* clinical isolates and their antibiotic resistance profile.

Strain designation	MIC (%)	MBC (%)	MBC/MIC	Antibiotic resistance pattern^∗^	Amp MIC (*μ*g/ml)	Amp MBC (*μ*g/ml)
101-001	0.260	0.391	1.5	Amp	0.25	2
101-380	0.326	0.781	2.4	Amp	0.25	2
102-102	0.521	0.781	1.5	Amo, Amp, Cham, Do, Enf, Gent	0.5	4
102-231-1	0.163	0.651	4	Amp, Cham, Do, Gent	0.25	2
100-605	0.195	0.391	2	Amo, Amp, Cef, Cep, Cham, Do, Enf, Gent	0.5	2

^∗^Amo, amoxicillin; Amp, ampicillin; Cef, cefazolin; Cep, cephalexin; Cham, chloramphenicol; Do, doxycycline; Enf, enrofloxacin; Gent, gentamicin.
